# Sleep Time Estimated by an Actigraphy Watch Correlates With CSF Tau in Cognitively Unimpaired Elders: The Modulatory Role of APOE

**DOI:** 10.3389/fnagi.2021.663446

**Published:** 2021-08-02

**Authors:** Sara López-García, Carmen Lage, Ana Pozueta, María García-Martínez, Martha Kazimierczak, Andrea Fernández-Rodríguez, María Bravo, Luis Reyes-González, Juan Irure, Marcos López-Hoyos, Eloy Rodríguez-Rodríguez, Pascual Sánchez-Juan

**Affiliations:** ^1^Cognitive Impairment Unit, Neurology Service and Centro de Investigación Biomédica en Red sobre Enfermedades Neurodegenerativas (CIBERNED), ‘Marqués de Valdecilla’ University Hospital, Institute for Research ‘Marqués de Valdecilla’ (IDIVAL), University of Cantabria, Santander, Spain; ^2^Photonics Engineering Group, University of Cantabria, Santander, Spain; ^3^Department of Immunology, ‘Marqués de Valdecilla’ University Hospital, Institute for Research ‘Marqués de Valdecilla’ (IDIVAL), Santander, Spain

**Keywords:** neurodegenerative diseases, Alzheimer’s disease, sleep disorder, amyloid, tau protein, actigraphy

## Abstract

There is increasing evidence of the relationship between sleep and neurodegeneration, but this knowledge is not incorporated into clinical practice yet. We aimed to test whether a basic sleep parameter, as total sleep estimated by actigraphy for 1 week, was a valid predictor of CSF Alzheimer’s Disease core biomarkers (amyloid-β-42 and –40, phosphorylated-tau-181, and total-tau) in elderly individuals, considering possible confounders and effect modifiers, particularly the *APOE ε*4 allele. One hundred and twenty-seven cognitively unimpaired volunteers enrolled in the Valdecilla Study for Memory and Brain Aging participated in this study. Seventy percent of the participants were women with a mean age of 65.5 years. After adjustment for covariates, reduced sleep time significantly predicted higher t-tau and p-tau. This association was mainly due to the *APOE* ε4 carriers. Our findings suggest that total sleep time, estimated by an actigraphy watch, is an early biomarker of tau pathology and that *APOE* modulates this relationship. The main limitation of this study is the limited validation of the actigraphy technology used. Sleep monitoring with wearables may be a useful and inexpensive screening test to detect early neurodegenerative changes.

## Introduction

Sleep disturbances are very common in Alzheimer’s disease (AD) and represent a major clinical problem (Pollak and Perlick, 1991; Moran et al., 2005). These symptoms appear early, and individuals with prodromal AD frequently have pronounced changes in sleep architecture and reduced sleep time (D’Rozario et al., 2020). Moreover, increasing evidence supports that sleep changes might be present in preclinical AD (Yaffe et al., 2014; Leng et al., 2019). A recent meta-analysis showed that sleep duration was the most consistent non-cognitive predictor of cognitive deterioration (Hudon et al., 2020).

A bidirectional link between chronic sleep disturbances and AD has been formulated (Ju et al., 2014). Animal and human experiments demonstrate the accumulation of amyloid-β (Aβ) after sleep deprivation (Lucey and Holtzman, 2015; Lucey et al., 2018). On the other hand, Aβ deposition perturbs the sleep-wake cycle (Lucey and Holtzman, 2015). Therefore, disrupted sleep would lead to the accumulation of amyloid in the brain which in turn would affect sleep, establishing a vicious circle that enhances the amyloid accumulation, increasing the risk of dementia (Ju et al., 2014). However, sleep decline is almost universal in elders (Ohayon et al., 2004), and to clarify its role as a clinical biomarker it is important to understand to what extent sleep disturbance is related to early signs of neurodegeneration. One way to address this issue is to correlate sleep parameters with pathophysiology proxies, such as Aβ or tau protein, the two hallmarks of AD, in cognitively healthy individuals. Several studies in elders have found associations between sleep and Aβ, determined by CSF levels or more frequently by PET scan (Ju et al., 2013; Spira et al., 2014; Sprecher et al., 2015; Branger et al., 2016; Brown et al., 2016; Varga et al., 2016). More recently, studies have explored the relationship between tau and sleep (Kam et al., 2019; Lucey et al., 2019). The majority of these studies have used self-reported questionnaires to evaluate sleep (Spira et al., 2014; Sprecher et al., 2015; Branger et al., 2016; Brown et al., 2016). Less frequently, sleep was assessed by one-night in-lab polysomnography (PSG) (Varga et al., 2016).

We are still far from incorporating sleep to AD risk prediction. First, we need more studies to clarify the relationship between the AD core biomarkers and sleep. In such studies, it is important to use accurate and objective sleep measurements but at the same time, these measurements should be scalable to allow for broad clinical use. Another essential point is to take into consideration possible effect modifiers or confounders, as the underlying mechanisms are poorly understood and might be modified by such factors as sex, depression symptoms, medication use, and very importantly, by the *APOE ε*4 allele. Several publications have reported that *APOE ε*4 affects sleep in cognitively healthy individuals (Drogos et al., 2016; Kahya et al., 2017) and that it might also play a modulatory effect between sleep and AD pathology (Lim et al., 2013; Burke et al., 2016; Hwang et al., 2018).

We hypothesized that sleep is associated with early neurodegenerative changes in cognitively healthy elderly individuals and that *APOE* might modulate this relationship. Therefore, we aimed to test whether total sleep time, measured by actigraphy during 1 week, was related to CSF AD core biomarkers (Aβ42/40, phosphorylated-tau-181, and total-tau) in elderly individuals, considering possible confounders and effect modifiers. We specifically aimed to explore the potential interaction effect between the *APOE ε*4 allele and sleep on AD pathology.

## Materials and Methods

### Participants

One hundred and twenty-seven volunteers enrolled in the Valdecilla Study for Memory and Brain Aging participated in this study. This is a prospective cohort recruiting community-dwelling non-demented people older than 55 years from the region of Cantabria (North Spain). Participants are phenotyped extensively and biological samples are obtained at baseline. This includes CSF analysis of AD core biomarkers and a comprehensive neuropsychological battery which is designed to detect early signs of AD and comprises several memory tests: the Free and Cued Selective Reminding Test (FCSRT) (Buschke, 1984), the Spanish version of the Face-Name Associative Memory Exam (S-FNAME) (Alegret et al., 2015), and the Logical Memory Test of the Wechsler Memory Scale-III (WMS-III LM) (Wechsler, 1945). Depression symptoms were also assessed by the Geriatric Depression Scale (GDS) (Sheikh and Yesavage, 1986). Only those individuals cognitively unimpaired, with a Clinical Dementia Rating (CDR) of 0 were included for this analysis (Morris, 1991).

### Sleep Assessment

Sleep monitoring was carried out over seven nights with actigraphy using a commercial activity wristband (Xiaomi Mi Band 2). The sleep parameter recorded was the average of total sleep time. The Mi Band 2 detects movements *via* a triaxial accelerometer. After a week, the device was connected with the researchers’ smartphones *via* Bluetooth, and a dedicated application (MiFit app) displayed the number of steps, wake up-times, bedtimes, as well as total sleep times for each day. Total sleep time was automatically measured, and it was calculated as the time interval between bedtime and wake-up-time having excluded periods of detected wakefulness (Topalidis et al., 2021). Sleep was also assessed by the Oviedo Sleep Questionnaire, a self-reported test validated for the Spanish population that provides scores for insomnia and sleep quality (Bobes et al., 2000). Sleep apnea diagnosis and sleep medications were reviewed in the clinical records.

### Biomarker Studies and *APOE* Test

The CSF biomarker assessment included the determination of amyloid-β-42 (Aβ42), amyloid-β-40 (Aβ40), total-tau (t-tau), and phosphorylated-tau-181 (p-tau). The lumbar puncture was performed on all participants from 9 AM to 10 AM. The levels of biomarkers were quantified by chemiluminescent enzyme-immunoassay (Lumipulse G600 II, Fujirebio Europe, Belgium) following the manufacturer’s instructions and interpreted according to the previously established cut-off points using amyloid PET as gold standard (Alcolea et al., 2019). To adjust for individual differences in total amyloid production, Aβ42 was expressed relative to Aβ40. *APOE* was genotyped using TaqMan SNP Genotyping Assays (Applied Biosystems, Foster City, CA, United States). Participants with ≥1 copy of the ε4 allele were considered ε4+. All others were considered ε4−.

### Statistical Analysis

Total sleep time was correlated with the three core biomarkers of AD (Aβ 42/40, t-tau, and p-tau) using Pearson’s r. Additionally, we used ANOVA and Tuckey’s *post hoc* test to compare sleep time across the “ATN” NIA-AA classification (Jack et al., 2018). Thus, individuals were categorized according to their biomarker’s status of Aβ deposition (A: Aβ), pathologic tau (T: p-tau) and neurodegeneration (N: t-tau).

We analyzed by Chi^2^ and Student’s *t*-test potential confounders or modifiers as sleep apnea, time from sleep assessment to lumbar puncture, depression symptoms (measured by the GDS), and use of sedative drugs. Multivariate analyses using General Lineal Models (GLM), with the CSF markers as dependent variables and average total sleep for 1 week, as the main predictors were performed. Age, sex, and *APOE ε*4 status were included as covariates in all models.

We assessed the potential synergistic effect between total sleep and *APOE ε*4 status by testing the interaction terms in the models. Further, stratified analyses by *APOE ε*4 status were performed.

A secondary aim was to explore the potential clinical use of sleep in AD diagnosis: (1) we correlated the main sleep questionnaire outputs (insomnia score and subjective impression of sleep quality) with CSF AD biomarkers, using Pearson’s r, to assess its clinical utility in comparison to the objective actigraphy data; (2) to test the hypothesis that sleep disturbances are early markers of degeneration we correlated, by Pearson’s r, total sleep time with the main outputs of three sensitive memory tests to detect early signs of AD (FNAME. FCSRT and LM).

All statistical analyses were performed with SPSS (Statistical Package for Social Sciences, 19).

### Ethics

The study was approved by the local Ethics Committee (reference number 2018.111) and all participants gave their written informed consent before participating in the study.

## Results

Out of the 127 participants, there were 70.1% females, and the mean age was 65.5 ± 6.3. The distribution of education levels was 34.5% tertiary, 38.8% secondary, and 26.7% primary. The average MMSE was 28.8 ± 1.5, and the FCSRT delayed cued recall score 14.8 ± 2 out of 16. The proportion of *APOE ε*4 carriers was 30.7%, and 37.8% had at least one positive AD core CSF biomarker. The average total sleep was 442.24 ± 73 min (7.37 h). According to the “ATN” classification, 62.2% of the individuals had all three biomarkers negative and 29.1% were in the Alzheimer’s continuum: 12.6% have amyloid positive biomarkers and p-tau and t-tau negative, and 16.5% had all three biomarkers positive. Three individuals were amyloid positive, p-tau positive and t-tau negatives, and only one individual was amyloid positive, p-tau negative and t-tau positive; all of them had borderline values for both tau markers, thus, these four individuals were pooled with those with all three positive markers. Finally, 8.7% were t-tau positive in the absence of Aβ (Table 1).

**TABLE 1:** Participants’ characteristics.

Characteristic	*n* = 127
Females, no. (%)	89 (70.10)
Age, mean (SD)	65.47 (6.33)
*APOE* ε4 carrier, no. (%)	39 (30.70)
**Education level**	
Tertiary no. (%)	40 (34.5)
Secondary no. (%)	45 (38.8)
Primary no. (%)	31 (26.7)
MMSE (0–30), mean (SD)	28.76 (1.49)
**Free and cued selective reminding test (FCSRT)**	
Free recall (0–16), mean (SD)	9.42 (3.65)
Cued recall (0–16), mean (SD)	14.76 (1.97)
Hypnotic drugs users once a week minimum, no. (%)	40 (31.49)
Geriatric depression scale (0–30), mean (SD)	6.34 (4.89)
Sleep Apnea-hypopnea syndrome diagnosis, no. (%)	16 (12.60)
**Oviedo Sleep Questionnaire:**	
Subjective sleep quality impression (0–7), mean (SD)	4.32 (1.67)
Insomnia score (0–45), mean (SD)	18.37 (6.83)
Sleep during the observational period:	
Average daily total sleep in minutes, mean (SD)	442.24 (72.99)
**CSF biomarkers:**	
Aβ40, mean (SD), pg/ml	10524.28 (3222.79)
Aβ42, mean (SD), pg/ml	795.72 (329.49)
Ratio Aβ42/40, Mean (SD)	0.076 (0.021)
Total-tau, mean (SD), pg/ml	337.92 145.73)
Phosphorylated-tau, mean (SD), pg/ml	45.06 (28.37)
**Alzheimer’s pathology, no. (%)**	
Amyloid neg/Tau neg	79 (62.20)
Amyloid neg/Tau pos	11 (8.66)
Amyloid pos/Tau neg	16 (12.59)
Amyloid pos/Tau pos	21 (16.53)
Time interval from sleep study-lumbar puncture in days, mean (SD)	144.72 (50.17)

*SD, standard deviation; Aβ, amyloid-β, neg, negative; pos, positive.*

### Sleep Correlated With CSF Tau

**FIGURE 1:**
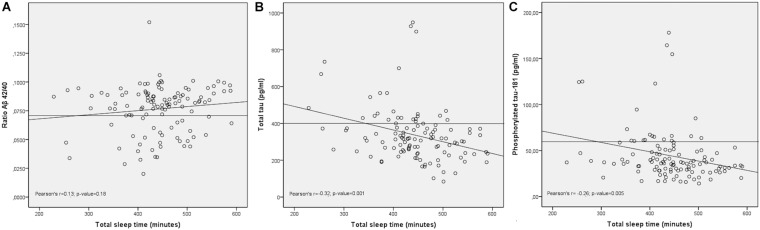
Relationship between total sleep with CSF AD biomarkers. Scatter plot showing the distribution of values for total sleep time (*X*-axis) and the Alzheimer’s biomarkers (*Y*-axis). The horizontal lines show cut-off points for each biomarker. The diagonal lines represent best fit of the linear equation. The correlations between the Aβ42/40 ratio with total sleep **(A)** was non-statistically significant. In contrast, total tau was negatively correlated with total sleep time **(B)**. Similarly, phosphorylated tau was negatively correlated with total sleep time **(C)**.

**FIGURE 2:**
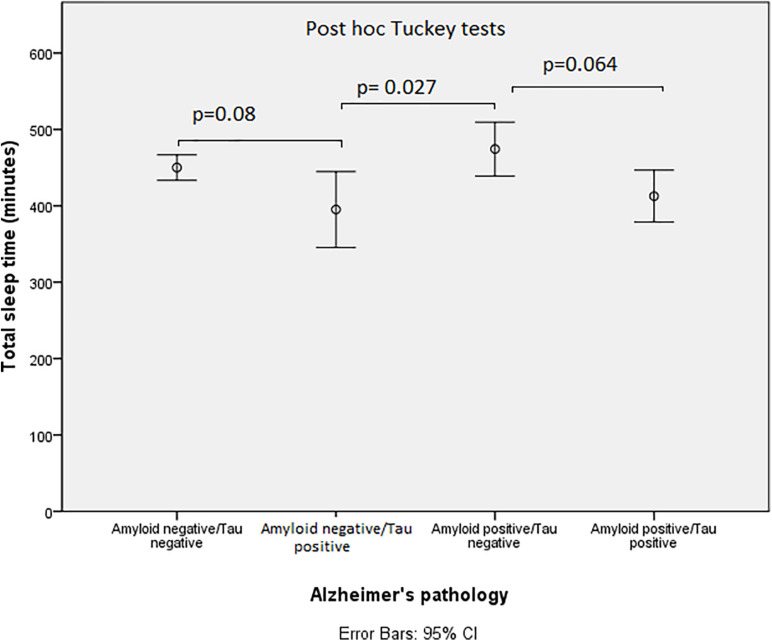
Distribution of total sleep time across the “ATN” NIA-AA classification. Error bars displaying total sleep time across the ATN groups show that individuals with positive tau biomarkers tended to have less sleep time.

We found an inverse correlation between total sleep time and CSF levels of t-tau (*r* = −0.32; *p* = 0.001) and p-tau (*r* = −0.26; *p* = 0.005); however, no significant association was found with Aβ42/40 (Figure 1). From another perspective, Figure 2 shows that individuals with positive tau biomarkers tended to have less sleep time, and that trend was also present in those individuals with suspected non-amyloid pathology (amyloid negative and t-tau positive).

### After Adjustment by Covariates, Sleep Parameters Predicted CSF Tau Levels

We found that neither the time between sleep assessment and lumbar puncture, sleep apnea diagnosis, treatment with hypnotics nor geriatric depression scale were associated with CSF markers. Therefore, these variables were not included in the model (Supplementary Table 1). When adjusted by age, sex, and *APOE ε*4 status, we found that total sleep was associated with t-tau (β = −0.96; *p* = 0.00036) and p-tau (β = −0.14; *p* = 0.0072). No association was found with amyloid markers (Table 2).

**TABLE 2:** Relationship between sleep and CSF AD biomarkers after adjusting for age, sex, and *APOE*4 status.

CSF biomarker	Sleep variable	Co-variates	Interaction	Beta	95% CI	*p*-value
Aβ 42/40	Average daily total sleep			0.000034	−0.000042 to 0.00011	0.37
		Age		−0.00073	−0.0013 to −0.00020	**0.0079**
		*APOE* ε4 non-carrier vs. carrier		0.040	−0.0024 to 0.083	0.060
		Females vs. males		0.0026	−0.0053 to 0.011	0.51
			Total sleep × *APOE* ε4	−0.000050	−0.00015 to 0.000047	0.31
T-Tau	Average daily total sleep			−0.96	−1.48 to −0.45	**0.00036**
		Age		8.91	5.23 to 12.58	**0.0000050**
		*APOE* ε4 non-carrier vs. carrier		−386.72	−677.59 to −95.85	**0.0096**
		Females vs. males		−16.84	−71.26 to 37.58	0.54
			Total sleep × *APOE* ε4	0.78	0.12 to 1.44	**0.021**
P-Tau-181	Average daily total sleep			−0.14	−0.24 to −0.039	**0.0072**
		Age		1.70	0.98 to 2.42	**0.0000079**
		*APOE* ε4 non-carrier vs. carrier		−62.17	−119.03 to −5.30	**0.032**
		Females vs. males		−6.53	−17.17 to 4.11	0.23
			Total sleep × *APOE* ε4	0.12	−0.0093 to 0.25	0.069

Aβ, amyloid-β; CI, confidence interval. *Statistically significant values are shown in bold.

### *APOE* Modulated the Effect of Sleep on CSF Tau Levels

**FIGURE 3:**
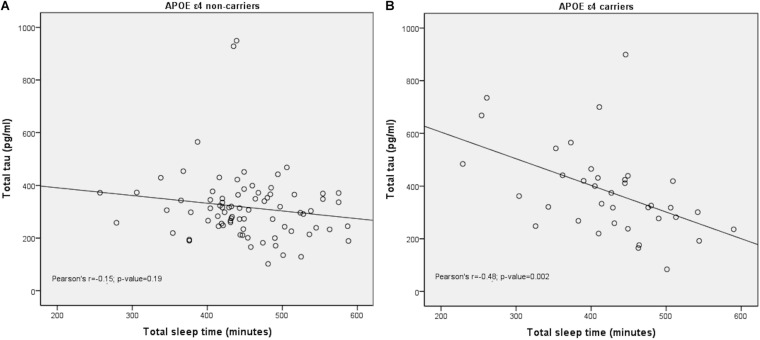
Distribution of total sleep time and total-tau levels in CSF stratified by *APOE*. Scatter plot showing the distribution of values for total sleep time (*X*-axis) and the tau levels (*Y*-axis) stratified by *APOE* ε4 status. While *APOE* ε4 non-carriers showed no correlation with total sleep **(A)**, *APOE* ε4 showed a negative correlation with total sleep **(B)**.

The interaction term between total sleep time and *APOE ε*4 was statistically significant in the GLM to predict t-tau levels (β = 0.78; *p* = 0.021) (Table 2). In Figure 3, the stratified analysis by *APOE ε*4 shows that the correlation between total sleep time with t-tau is mainly due to the *APOE ε*4 carriers. Additionally, A*POE ε*4 carriers had less total sleep time than non-carriers (422.3 ± 81.6 vs. 451.57 ± 67.13 min, respectively; *p*-value = 0.044).

### Self-Reported Assessment of Sleep Did Not Correlate With AD Biomarkers

Supplementary Table 2 shows that neither perceived sleep quality nor insomnia scores from the Oviedo Sleep Questionnaire did correlate with any of the AD biomarkers.

### Total Sleep Time Did Not Correlate With Memory Tests in Our Cognitively Healthy Population

In our population, we found no association between any of the memory test results and the total sleep time (Supplementary Table 3).

## Discussion

The most important finding of our study was the inverse correlation between total sleep time estimated by a commercial actigraphy watch and tau pathology in a cognitively healthy population. Our results are in line with previous observational studies linking sleep duration with cognitive impairment and dementia risk (Yaffe et al., 2014). These studies have been recently reviewed in a meta-analysis showing that both short and long sleep duration was consistently associated with worse cognitive performances (Hudon et al., 2020). Therefore, it has been proposed that the association between sleep time and the risk of cognitive deterioration is V-shaped. However, the fact that most of these studies employed self-reported assessments of sleep time, and evaluated individuals at different stages in the AD continuum, may have contributed to some inconsistency in the results (Tworoger et al., 2006; Schmutte et al., 2007; Faubel et al., 2009; Blackwell et al., 2011; Ramos et al., 2013; Yaffe et al., 2014). Our analysis using objective estimation of total sleep and core AD CSF biomarkers as a proxy for dementia risk, strongly supports that those cognitively healthy elderly individuals with lower sleep time are potentially at greater risk of future neurodegenerative diseases based on higher t-tau and p-tau levels in the CSF.

Despite the evidence of the role of Aβ as a potential link between sleep and AD, we were not able to find an association with Aβ CSF levels. Most studies analyzing sleep and Aβ have used amyloid PET and employed self-reported sleep assessments (Spira et al., 2014; Sprecher et al., 2015; Branger et al., 2016; Brown et al., 2016; Carvalho et al., 2018); finding statistical associations with a variety of sleep-related variables like sleep latency (Branger et al., 2016; Brown et al., 2016), daytime sleepiness (Carvalho et al., 2018), or self-reported less adequate sleep (Sprecher et al., 2015, 2017); only Spira et al. (2014) found an association with sleep time, but was self-reported as well. The use of CSF Aβ analysis instead of amyloid PET might also explain differences in results. Even though it has been shown that CSF amyloid levels (Aβ40 and Aβ42) increase in humans after sleep deprivation (Lucey et al., 2018), we must consider that in preclinical AD Aβ42 levels decreases in comparison to Aβ40. To our knowledge, our study includes the largest sample assessing the relationship between an objective estimation of sleep time and Aβ in CSF in cognitively healthy individuals. In line with our results, those studies that used actigraphy to determine sleep time objectively did not find an association with Aβ (Ju et al., 2014; Lucey et al., 2019). On the other hand, perhaps a determination of sleep components, such as slow-wave activity (SWA), might have increased our chances to detect an association with Aβ, as reported before (Varga et al., 2016; Kam et al., 2019; Liguori et al., 2020), although a recent study, with a sample size similar to ours, did not find an association between SWA and Aβ levels (Lucey et al., 2019).

Our data would suggest an early association between tau and sleep independent of Aβ in cognitively healthy individuals. Prospective studies using PET support that tau and amyloid, initially have separate trajectories, although at some point they interact and amyloid drives tau extension beyond the medial temporal lobes (Schöll et al., 2016). Therefore, in AD the deposits of these two proteins follow different patterns, and contrary to the Aβ pathology that usually starts in the cortex, tau accumulates first in the brainstem and subcortical regions (Serrano-Pozo et al., 2011). Recent neuropathological studies have shown that subcortical brain areas implicated in sleep-wake regulation, are very vulnerable and early affected by AD tau neurodegeneration (Oh et al., 2019). In a longitudinal study, it was found that uninterrupted sleep was associated with less neurofibrillary tangles load in the *APOE ε*4 carriers, but there was no association with Aβ pathology (Lim et al., 2013). Finally, our study is in line with recent publications showing the association between tau pathology and several sleep neurophysiological parameters like SWA (Lucey et al., 2019) and the density of spindles at non-rapid eye movements (NREM) stage 2 (Kam et al., 2019). Therefore, sleep changes in individuals at preclinical stages of AD might correlate with early tau brainstem pathology, and as a sort of “canary in a coal mine,” sleep disturbances might be an early AD biomarker (Iranzo et al., 2009; Bombois et al., 2010). Alternatively, chronic poor sleep might be causing higher tau levels and potentially increasing the risk of neurodegeneration. The same research group that has pioneered studies linking amyloid dynamics to sleep has recently discovered that tau levels are also strongly influenced by the sleep-wake cycle. In mice, tau levels in interstitial fluid and CSF increased around 90% during wakefulness and 100% during sleep deprivation. In human CSF tau increased 50% after sleep deprivation. And these findings appeared to have pathological significance, as in a tau seeding and spreading mouse model chronic sleep deprivation increased tau pathology spreading (Holth et al., 2019). Nevertheless, both mechanisms are compatible, and there could also be a two-way association between tau and sleep.

Our second main finding was the significant interaction between sleep and *APOE ε*4 carriers, which together with the stratified analysis indicates that the relationship between sleep and tau was predominantly due to the *APOE ε*4 individuals. This is in line with recent publications indicating that ApoE plays a fundamental role in tau pathology. It has been demonstrated that ApoE affects tau pathogenesis, neuroinflammation, and tau-mediated neurodegeneration independently of Aβ pathology (Shi et al., 2017). Additionally, Koch and colleagues have shown that only in *APOE* ε4 AD patients there is a strong association between cognitive decline, impaired cortical plasticity and CSF t-tau values, suggesting a relative susceptibility to *APOE ε*4 patients to the amount of tau-related pathological burden (Koch et al., 2017). Our results are in line with previous studies suggesting the role of *APOE* as a modulator of the relationship between sleep and AD pathology. In individuals with the *APOE ε*4 allele, less sleep consolidation was related to higher AD incidence and tau pathology load in the autopsy (Lim et al., 2013). We also found that, as reported before (Drogos et al., 2016), carriers of *APOE ε*4 slept significantly less time than non-carriers. As a potential explanation, it has been proposed that *APOE ε*4 individuals might have more respiratory problems during sleep (Gottlieb et al., 2004), supporting this hypothesis, several studies have found higher levels of tau pathology in sleep apnea patients (Liguori et al., 2017; Carvalho et al., 2020). Additionally, cognitively healthy *APOE ε*4 carriers had lower gray matter volume if they suffered from insomnia (Grau-Rivera et al., 2020). In our study, we did not find an association between CSF biomarkers and sleep apnea; however, we only assessed sleep respiratory problems based on clinical records.

### Limitations

In cross-sectional designs, biases are more likely, and confounders more difficult to disentangle. To minimize these threats, we used objective measurements of our two main variables: actigraphy for sleep and CSF markers for AD neurodegeneration. Additionally, we have considered the main possible confounders discussed in previous articles, performing a multivariate analysis to adjust for the main factors related to both sleep and CSF markers, and analyzing the synergistic role of the *APOE ε*4 allele. Another classic limitation of cross-sectional designs is that it is difficult to establish causality. This is an especially difficult question given the potential bi-directional relationships between sleep and neurodegeneration. Longitudinal studies would be essential to clarify this issue, though, our data offer some hints that sleep is an early marker. For instance, the absence of a significant correlation with the tests assessing memory in our population support that sleep disturbances precede detectable cognitive problems. A technical limitation is that our sleep assessment method was not able to delineate the sleep macrostructure. We acknowledge this is an important limitation, as some specific sleep periods, most significantly SWA, that we were not able to quantify individually might be more sensitive and specific to detect neurodegenerative changes, especially Aβ (Ju et al., 2017). Another limitation is the paucity of published data validating this particular model of wristband to estimate sleep. In a validation study with 27 healthy sleepers the overall agreement between PSG and the Mi band 2 was 84.69% to discriminate wake from sleep. However, they found that the Mi band 2 tended to overestimate the total sleep time (on average 69 min), an observation that has been also reported by a posterior study (Topalidis et al., 2021). In this same article they as well criticize the lack of reliability of several wristbands models to assess sleep architecture (Ameen et al., 2019). This is of course an important limitation, as the accuracy of sleep time estimation might not be ideal. However, the objective of this study is to provide proof of principle that even low-cost devices may help correlating basic sleep measurements with neurodegeneration markers.

## Conclusion

Our study has explored the clinical applicability of sleep changes as potential early biomarkers of neurodegeneration. Our data suggest that a simple sleep parameter, such as total sleep time estimated by an actigraphy watch, is a valid predictor of tau pathology with potential clinical use. In contrast, self-assessment with a sleep questionnaire did not yield any significant association with AD CSF biomarkers. These devices allow complex and variable data to be monitored over long periods, enabling more ecological observation than laboratory assessments, as PSG, which can compensate for the lower accuracy of the estimate. Importantly, this is an inexpensive and scalable technology that could be incorporated to risk prediction algorithms together with other readily available information. However, studies tracking sleep changes during more prolonged periods, and the use of more accurate portable devices that provide information about the architecture of sleep, could significantly improve the diagnostic performance of sleep assessment as an AD screening method. Longitudinal studies may also help to understand better the direction of this association and the potential value of preventive measures.

## Data Availability Statement

The original contributions presented in the study are included in the article/Supplementary Material, further inquiries can be directed to the corresponding author/s.

## Ethics Statement

The studies involving human participants were reviewed and approved by the Comité de Ética de Investigación Clínica de Cantabria (CEIC). The patients/participants provided their written informed consent to participate in this study.

## Author Contributions

SL-G: collection of the clinical data and drafting of the manuscript. CL: collection of the clinical data and critical revision of the manuscript. AP and MG-M: neuropsychological assessments. MK, AF-R, and MB: collection of the clinical data. LR-G: collection of the sleep data. JI and ML-H: CSF analytical procedures. ER-R: critical revision of the manuscript. PS-J: study concept and design, data analysis, drafting of the manuscript, and funding acquisition. All authors contributed to the article and approved the submitted version.

## Conflict of Interest

The authors declare that the research was conducted in the absence of any commercial or financial relationships that could be construed as a potential conflict of interest.

## Publisher’s Note

All claims expressed in this article are solely those of the authors and do not necessarily represent those of their affiliated organizations, or those of the publisher, the editors and the reviewers. Any product that may be evaluated in this article, or claim that may be made by its manufacturer, is not guaranteed or endorsed by the publisher.
