# Supplementary material for: Sleep Time Estimated by an Actigraphy Watch Correlates With CSF Tau in Cognitively Unimpaired Elders: The Modulatory Role of APOE
Source: Front Aging Neurosci. 2021 Aug 2;13:663446. doi: 10.3389/fnagi.2021.663446 (PMC8366270; doi:10.3389/fnagi.2021.663446)
Supplement: Supplementary file 1 [file Data_Sheet_1.docx]

**Table e-1: Participants´ characteristics according to CSF biomarker's status.**

**Table e-2: Correlation between Oviedo Sleep questionnaire main variables and Alzheimer's Disease biomarkers levels in CSF.**

**Table e-3: Correlation between memory tests and total sleep time.**

**Table e-1: Participants´ characteristics according to CSF biomarker's status.**

| Characteristics | Aβ42/40 positive | Aβ42/40 negative | p-value | T-tau positive | T-tau negative | p-value | P-tau positive | P-tau negative | p-value |
| --- | --- | --- | --- | --- | --- | --- | --- | --- | --- |
| Females, No. (%) | 23 (62.2) | 66 (73.3) | 0.21 | 14 (50.0) | 75 (75.8) | **0.0090** | 13 (50.0) | 76 (75.2) | **0.012** |
| Age, Mean (SD) | 67.7 (5.0) | 64.6 (6.6) | **0.012** | 69.5 (6.5) | 64.3 (5.8) | **0.000093** | 69.5 ()5.2 | 64.4 (6.2) | **0.00020** |
| APOE ɛ4 carriers, No. (%) | 23 (62.2) | 16 (17.8) | **0.00000083** | 16 (57.1) | 23 (23.2) | **0.0010** | 15 (57.7) | 24 (23.8) | **0.0010** |
| Geriatric Depression Scale, Mean (SD) | 7.6 (5.2) | 5.8 (4.5) | 0.051 | 6.3 (4.1) | 6.3 (4.9) | 0.99 | 6.34(4.4) | 6.3 (4.8) | 0.93 |
| Time between sleep assessment and lumbar puncture, Mean (SD) | 140.65 (36.35) | 149.05 (55.15) | 0.44 | 144.10 (38.98) | 147.29 (53.08) | 0.80 | 141.95 (38.78) | 147.73 (52.93) | 0.65 |
| Hypnotic drugs users once a week minimum, No. (%) | 11 (29.7) | 29 (32.2) | 0.78 | 5 (17.9) | 35 (35.4) | 0.078 | 7(26.9) | 33 (32.7) | 0.57 |
| Sleep Apnea-hypopnea syndrome, No. (%) | 3 (8.1) | 13 (14.4) | 0.33 | 3 (10.7) | 13 (13.1) | 0.73 | 3 (11.5) | 13 (12.9) | 0.73 |
| Average daily Total Sleep in minutes, Mean (SD) | 440.8 (72.4) | 442.83 (73.7) | 0.89 | 400.3 (72.8) | 453.8 (69.1) | **0.0010** | 403.7 (63.8) | 451.8 (72.3) | **0.0040** |

**Table e-2: Correlation between Oviedo Sleep questionnaire main variables and Alzheimer's Disease biomarkers levels in CSF.**

|  | Aβ42/40 levels  Pearson's r (p-value) | T-tau levels  Pearson's r (p-value) | P-tau levels  Pearson's r (p-value) |
| --- | --- | --- | --- |
| Subjective sleep quality impression | 0.027 (0.76) | 0.068 (0.45) | 0.046 (0.61) |
| Insomnia score | 0.048 (0.59) | -0.15 (0.10) | -0.087 (0.33) |

Abbreviations: Aβ, amyloid-β.

**Table e-3: Correlation between memory tests and total sleep time.**

|  | Total Sleep time  Pearson's r (p-value) |
| --- | --- |
| S-FNAME total (0-96) | -0.043 (0.62) |
| WMS-III LM Delayed Recall Units (0-50) | 0.075 (0.39) |
| FCSRT delayed free recall (0-16) | 0.117 (0.18) |
| FCSRT delayed cued recall (0-16) | 0.106 (0.22) |

Abbreviations: S-FNAME, Spanish version of the Face*-*Name Associative Memory Exam; WMS-III LM, Weschler Memory Scale-III Logical Memory subtest; FCSRT, Free and Cued Selective Reminding Test.
